# Adult Neurogenic Lower Urinary Tract Dysfunction and Intermittent Catheterisation in a Community Setting: Risk Factors Model for Urinary Tract Infections

**DOI:** 10.1155/2019/2757862

**Published:** 2019-04-02

**Authors:** Michael Kennelly, Nikesh Thiruchelvam, Márcio Augusto Averbeck, Charalampos Konstatinidis, Emmanuel Chartier-Kastler, Pernille Trøjgaard, Rikke Vaabengaard, Andrei Krassioukov, Birte Petersen Jakobsen

**Affiliations:** ^1^Department of Urology, Carolinas Medical Center, Charlotte, NC, USA; ^2^Department of Urology, Addenbrooke's Hospital, Cambridge, UK; ^3^Moinhos de Vento Hospital, Porto Alegre, Brazil; ^4^Urology & Neuro-urology Unit, National Rehabilitation Center, Athens, Greece; ^5^Urology Clinic, Hôpital Raymond Poincaré, Paris, France; ^6^Coloplast A/S, Humlebaek, Denmark; ^7^Division of Physical Medicine and Rehabilitation, Department of Medicine, Faculty of Medicine, University of British Columbia, Vancouver, British Columbia, Canada; ^8^G.F. Strong Rehabilitation Centre, Vancouver, British Columbia, Canada; ^9^Independent Medical Consultant, MD, MedDevHealth, Copenhagen, Denmark

## Abstract

A risk factor model for urinary tract infections in patients with adult neurogenic lower urinary tract dysfunction performing clean intermittent catheterisation was developed; it consists of four domains, namely, (1) general (systemic) conditions in the patient, (2) individual urinary tract conditions in the patient, (3) routine aspects related to the patient, and (4) factors related to intermittent catheters *per se*. The conceptual model primarily concerns patients with spinal cord injury, spina bifida, multiple sclerosis, or cauda equina where intermittent catheterisation is a normal part of the bladder management. On basis of several literature searches and author consensus in case of lacking evidence, the model intends to provide an overview of the risk factors involved in urinary tract infections, with specific emphasis to describe those that in daily practice can be handled and modified by the clinician and so come to the benefit of the individual catheter user in terms of fewer urinary tract infections.

## 1. Introduction

Adult neurogenic lower urinary tract dysfunction (ANLUTD) refers to “abnormal or difficult function of the bladder, urethra (and/or prostate in men) in mature individuals in the context of clinically confirmed relevant neurologic disorder” [1] (Appendix).

For community, residing ANLUTD subjects, clean intermittent catheterisation (CIC) is the gold standard for bladder emptying, as CIC is the safest method (voluntary, complete, low pressure emptying procedure) in terms of having the lowest potential for urological complications [2]. Indwelling transurethral catheterisation and, to a lesser extent, suprapubic cystostomy should be avoided due to high risk of UTIs and for significant long-term complications [2–4].

CIC is carried out between 16 and 56% of a spinal cord injury (SCI) population, depending on age and severity [5, 6]. In multiple sclerosis (MS) of longer duration, 68%–75% have urinary dysfunction [7, 8]; continuous use of CIC is dependent on the individual's perception of improvement in symptoms versus the burden of use and the appearance of a urinary tract infection (UTI) may also negatively influence the use of CIC [9]. In (adult) spina bifida (SB) patients CIC is a cornerstone in bladder treatment [10].

From daily clinical experience, clinical studies, and surveys, it is evident that UTIs are the commonest complication to intermittent catheterisation (IC) and constitutes a major reason for concern in patients and their clinicians and care givers [11]. The reported UTI incidence rates varies; during the rehabilitation phase of a SCI patient, UTI incidence rates were between 2 and 10 per year [12–19], whereas the UTI rates in the community-based population (>12 months after injury) ranged from 0.8–3.5 per year [20–27]. The present review focusses on community related data.

Several models have been used to describe risk factors for UTI in ANLUTD; Shekelle et al. [28] looked at three domains, the person and their functional level, balanced storage phase, and method of bladder drainage. Bladder emptying methods and residual urine were determined as clear risk factors, whereas reliable evidence was insufficient or missing for other factors. The model presented by Vasudeva and Madersbacher [29] identified intrinsic defence mechanisms, impaired washout, and catheterisation as culprits and concluded that further research is needed to understand the complex interplay between these mechanisms.

**Figure 1:**
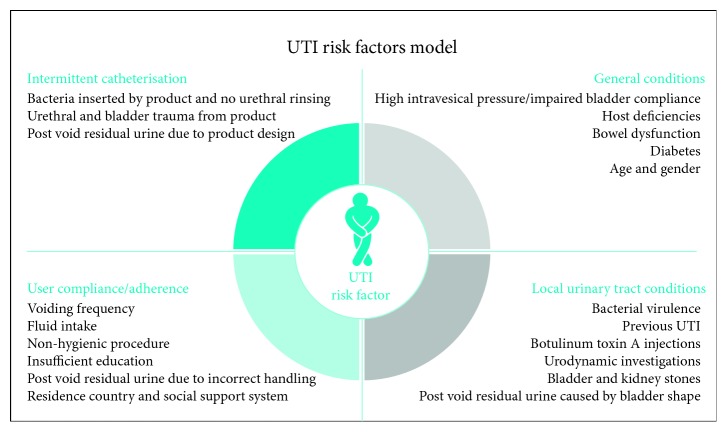
The UTI risk factors model with its four domains.

In a community-based setting of the ANLUTD population, the authors developed a simple, holistic clinical model (Figure 1) that encompasses four main areas of risk factors related to the neurogenic bladder patients performing IC, general (systemic) conditions, local urinary tract conditions, compliance, and intermittent catheters *per se*. The model should provide a useful tool to identify risk factors in the individual IC user.

## 2. Methods

At an initial workshop, upon a discussion of common risk factors for UTI in the IC users, the five experts, representing neurourology, rehabilitation medicine, and urology, expressed a need for a paper describing risk factors for UTI in the ANLUTD population; the model should aim, in a holistic and simple way, to visualise and describe the most common risk factors in this population and thereby provide a useful tool for health-care personal in the daily evaluation management of UTIs in connection with IC.

In a following workshop, inspired by the models of Shekelle et al. [28] and Vasudeva and Madersbacher [29], it was agreed that risk factors logically could be classified as belonging to one of four domains, namely, (1) general (systemic) conditions in the patient, (2) individual urinary tract conditions in the patient, (3) routine aspects related to the patient, and (4) factors related to intermittent catheters *per se* (Figure 1). The model should consider patients with SCI, SB, MS, and cauda equina (CE) where IC is a normal part of the bladder management. It was decided to describe the model in terms of a narrative review (position paper), as this approach appeared most relevant for the model in question.

Based on this workshop, two basic searches were performed; one search was performed in PubMed, Embase, and Cinahl using the same terms as in the original 1999 Shekelle et al.'s [28] review. Search: urinary tract, urinary tract infection, bacteriuria, paraplegia, quadriplegia, spinal cord injury, multiple sclerosis, neurogenic bladder, neuropathic bladder. Filters: Comparative Study, Controlled Clinical Trial, Meta-Analysis, Multicenter Study, Systematic Reviews, from 1999/08/01 to 2018/08/01, Humans, English, French, German, Adult: 19+ years. This retrieved 166, 37, and 3 citations in PubMed, Embase, and Cinahl, respectively. The citations in Cinahl and Embase were all included in the PubMed search. Nine relevant publications were identified [14, 17, 18, 20, 24, 30–33].

A further PubMed search was based on a modified Vasudeva search strategy: urinary tract infection, neurogenic bladder, spinal cord injury, spina bifida, multiple scleroses, cauda equina, bacterial flora, postvoid residual urine, reflux, intermittent catheterisation. Filters: from 1999/08/01 to 2018/08/01, Humans, English, French, German, Adult: 19+ years. This retrieved 8 citations, of which none described risk factors for UTIs. Additional literature searches for each specific risk factor (risk factor and UTI) were performed if deemed necessary.

All potential risk factors detected were presented at a third workshop. Using a modified Delphi approach, each risk factor was evaluated and discussed by the experts and, if found relevant for the model, classified to one of the four domains described above; in case of lacking or inconclusive evidence, the authors concluded by consensus. During this process, the model was finalised. The model intends to provide an overview of the risk factors involved, with specific emphasis to describe those that in daily practice can be handled and modified by the clinician and so come to the benefit of the individual IC user.

## 3. UTI: Definitions, Diagnoses, and Perceptions

### 3.1. UTI Definitions and Diagnoses

Uncomplicated UTI is a bacterial presence within the bladder and associated structures in patients with no structural abnormality and no comorbidities, whereas every UTI in patients with ANLUTD per definition are complicated. A key factor complicating the study of UTIs is the lack of consensus regarding its definition [33–35]. Different definitions of UTI for patients relying on IC not only consider laboratory parameters, but also signs and symptoms (Table 1). Among patients with neurourological diseases some present with impaired sensations in the lower urinary tract, like SCI subjects. They may find it difficult to report UTI-related symptoms accurately [36], which imposes a limitation to differentiate asymptomatic bacteriuria (AB) and UTI in the clinical practice. AB is a frequent finding in neurourological patients who perform IC, and it is a constant challenge to distinguish harmless colonization from pathogenic infection. According to international guidelines, prophylactic antibiotics should not be routinely prescribed to neurourological patients with AB, as this treatment might result in significantly more resistant bacterial strains without improving patient outcome [37, 38]. However, some studies have reported beneficial effect of prophylactic intervention [26, 39].

The controversy regarding UTI definitions was also described in a systematic review trying to more accurately examine the UTI rate following botulinum injections; they found that amongst 50 selected publications, only 27 had defined the UTI with a total of 10 different definitions [43]. Two studies used the National Institute on Disability and Rehabilitation Research [44] UTI definition, whereas no studies used a definition which met the European Association of Urology [4] or Infectious Diseases Society of America [41] criteria.

Biomarkers for UTI have been assessed in a limited number of trials assessed the role of biomarkers, such as interleukins 6 and 8, C-reactive protein, and erythrocyte sedimentation rate, for predicting UTI in humans. Whilst detection of urinary tract infection appears more sensitive with the use of urinary interleukins than that with traditional culture or dipstick techniques, the test is not widely available, is not a simple bedside test, and is more costly than a standard urine dipstick and urine culture testing [35].

Another aspect is the variability regarding definition of recurrent UTIs. Some studies consider 0-1 UTI/year representing no infection problems [45, 46], some differentiate between 0, 1-2 (infrequent), and 3 or more (recurrent) UTIs per year [25] and other consider 0–2 and 3 or more UTIs per year as sporadic and recurrent UTIs, respectively [47]. Whatever definition, a subpart of the population does consistently not experience UTIs when reported on a yearly basis. There may also be a difference in incidence rates between self-reported UTIs and those medically documented in retrospective and prospective studies [17, 20, 25]. Altogether, such heterogeneous UTI definitions seriously hamper the clinical and research efforts and may over- or underscore the importance of various risk factors.

### 3.2. Patient Perspective on UTIs

CIC is widely used for the urological management of neurogenic bladders, and UTI is the most frequently reported and challenging complication [48]. The diagnosis of UTI in the general population is not generally applicable to individuals with SCI, SB, or CE due to loss of sensation and a neurogenic bladder requiring alternative bladder emptying like CIC. The common symptoms include increased bladder spasticity, new or worsening urinary incontinence, autonomic dysreflexia, and foul-smelling urine.

The NIDRR consensus statement on the prevention and management of UTI among people with SCI listed a set of signs and symptoms as suggestive of UTI [44]. The validity, accuracy, and predictive value of these signs and symptoms were evaluated during the first 3 months of a 1-year-long randomised controlled CIC-catheter trial (RCT) in chronic SCI patients [20, 49]. Subjects were able to predict their own UTI with an accuracy of 66%, and with positive and negative predictive values of 33% and 83%, respectively. Okamoto [50], evaluating CIC-users in general, presented findings along the same line, underlining the CIC-user's insecurity regarding the interpretation of their UTI signs and symptoms. Self-reported UTIs should hence be interpreted with care.

### 3.3. Rehabilitation Centres' Perspective on UTIs

A questionnaire-based survey of 13 German-speaking SCI rehabilitation centres sheds light on the variability of handling the UTIs in SCI patients (irrespective of bladder management), both with respect to diagnosing as well as treating a UTI [51]. The criteria for accepting a UTI urine analysis as positive differed and the symptomatic treatment was started with “fever without other causes” as the most frequently symptom irrespective of leucocyte count. These finding underlines both the impact of the personal experience of the treating physician and the paucity of published evidence.

When evaluating the evidence on UTI risk factors in neurogenic CIC-users, issues like heterogenic UTI definitions, uncertainty of patients' self-reported data, and significant differences between medical evaluation and treatment of UTIs could profoundly influence the level of importance of the separate risk factors. Additionally, the importance of specific risk factors may differ between individual patients.

## 4. The Model: General Conditions

### 4.1. High Intravesical Pressure/Impaired Bladder Compliance

With any spinal cord injury, the disruption of the normal somatic and autonomic neurological control of bladder causes bladder dysfunction; in higher suprasacral injuries, a micturition reflex may emerge after the acute phase to cause detrusor overactivity and sometimes detrusor-sphincter dyssynergia (DSD) with ineffective bladder emptying and clinical significant large postvoid residual volumes. Lesions in the lumbosacral area affecting the autonomic nuclei may cause detrusor hypocontractility with bladder over-filling and voiding inability. All SCI and most of the SB patients have bladder dysfunction; it is common in MS, and if disease duration is above 10 years, up to 80% of the MS patients have bladder symptoms [11].

Individuals with high intravesical pressures and impaired bladder compliance are more prone to UTI's than those with low intravesical pressure and high capacity bladder that it is emptied periodically by IC. The risk factors are the high intravesical pressures in the first case and the risk of bladder over-distention in the other case [52].

The degree of bladder dysfunction (compliance, storage pressure), assessed through urodynamic parameters, appears to correlate to increased UTI incidence rates. In a retrospective study, low bladder compliance (<10 ml/cm H_2_O), detrusor overactivity, and vesicoureteral reflux correlated with increased UTI incidence rates in 76 spina bifida patients performing CIC [53]. In contrast, a recent retrospective study could not verify a correlation in 194 SB children [46]. Experience from clinical practice supports a correlation between UTIs and poorly compliant bladder.

It has been speculated that Bladder ischemia due to decreased blood flow predisposes to UTI [54, 55]. In the neurogenic bladder, this may happen in relation to (untreated) increased intravesical pressure and overdistension due to large urine volumes [29]. Lapides suggested that “it was logical to assume that maintenance of a good blood supply by prevention of vesical overdistension and elevated intravesical pressures would combat infection” [54]. A 7-year prospective study showed that a mean volume of each catheterisation >400 ml is linked UTI [45].

### 4.2. Host Deficiencies (Impaired Immune System)

Altered intrinsic defence mechanisms and immune suppression after spinal injuries augment the risk for UTIs possibly due to changes in the microbial flora, immunological deficiencies [56], and alterations in the bladder wall and its urothelium [29, 57]. Such conditions are presently not readily modifiable in daily clinical practice. Apart from the local injury in the spinal cord, patients may develop a variety of complications characterized by multiple-organ dysfunction such as lung injury, cardiovascular disease, liver and kidney damage, and increased susceptibility to infection. The damage to the autonomic nerve system (ANS) leads to a general immune dysfunction through the loss of neural innervation of lymphoid organs.

### 4.3. Bowel Dysfunction

The distal colon and urinary bladder have a similar function of the storage and evacuation of faeces and urine, and there is a joint peripheral innervation of both viscera, through the hypogastric, pelvic, and pudendal nerves [58]. It is therefore not unexpected that spinal cord injuries also affect colorectal motility, transit times, and bowel emptying, leading to constipation, faecal incontinence, or a combination of both. When treating neurogenic bowel dysfunction in terms of faecal incontinence and constipation with transanal irrigation, a more than three-fold reduction of UTI incidence rates was documented [59, 60]. The reason for this UTI reduction is unknown; it could be speculated that fewer episodes of faecal incontinence causes less genitourinary bacterial contamination or, given the role of the kidneys and bladder in filtration and storage of waste, respectively, microbial profiles and microbial metabolites of the gut might influence the urinary microbiota, and alterations might affect urinary homeostasis [61]. Further, rectal impaction has been suggested to cause lower urinary tract symptoms (LUTSs) by mechanically to impede bladder emptying [62]. Hence, an optimal bowel treatment of constipation and incontinence should go hand in hand with bladder management.

### 4.4. Diabetes

Diabetes *per se* bears a 2- to 3-fold risk for UTI compared to nondiabetic controls, and the severity is usually more severe and carries worse outcomes [63, 64]. A large cross-sectional Canadian Community Health Survey compared the prevalence of type 2 diabetes in the SCI population with that of a nonSCI population [65]. They found, regardless of the variables included in the models, an approximately 2-fold increased odd for type 2 diabetes in the SCI population, which is not explained by known risk factors for type 2 diabetes. In other words, unexplained occurrences of UTI may be related to undiagnosed diabetes.

### 4.5. Age and Gender

It is inconclusive whether age or gender plays a major role for the UTI risk in the neurogenic population; some studies suggest a slightly higher UTI rate for females [25, 45], whereas older studies reported conflicting results [28]. A recent, retrospective chart review of 194 SB patients (median age 22 years, range 8 months–58 years) found that increasing age was associated with decreasing odds of UTI by 7% per year, independent of gender [46]. As the authors could not verify compliance with the recommended CIC frequency or technique, age and maturity may have impacted the patient's ability to manage their bladder emptying and hence risk of UTI.

## 5. The Model: Local Urinary Tract Conditions

### 5.1. Bacterial Virulence

The development of UTIs in the neurogenic bladder relays on a balance between bacterial virulence and local host factors. When the ability to locally fight the infection is compromised, the uropathogens have better access to the urinary tract and their washout of the bladder is lost [29]. Further, upon alteration of protective flora and changes in the urothelium and bladder wall after injury may let the uropathogens inside the urinary tract more easily adhere to the urothelium and invade the bladder wall [57, 66]. All such events may end up in UTIs; however, investigation and treatment of these factors are at present on an experimental level.

Antibiotics may also interfere with the protective flora. A study in 70 women with UTIs showed that the original lactobacillus population had not been restored after treatment in most patients; rather, the uropathogenics dominated the flora [67]. A similar condition could be relevant for the microbiome in the bladder.

The strategy of supporting the host bacterial flora by adding nonpathogenic bacteria have been investigated in a couple of small studies [68, 69]. Inoculation of nonpathogenic bacteria (mostly *E. Coli*) into the bladder showed sufficient colonization rates of the inoculated pathogenic bacteria and significant reductions in UTI frequency. It appears that this approach could be useful, although there is still insufficient evidence to support the use of bacterial interference for UTI prevention in daily practice [4, 42].

### 5.2. Previous UTIs

Previous UTIs are accepted as a risk factor because they render the bladder urothelium into a chronic, inflammatory condition more susceptible to reinfection. A 7-year prospective study of CIC-users found two predictive factors; patients with high UTIs at the start of the study also had high UTI rates at the end of the follow-up period and high UTI rates were linked to development of high catheterisation volumes [45].

### 5.3. Botulinum Toxin A Injections

Detrusor treatment with Botulinum Toxin A injections efficiently combats neurogenic detrusor overactivity [70] but there is controversy about the UTI rates postinjection [43] and whether there should be antibiotic coverage for the therapeutic sessions [71]. Data from a single SCI rehabilitation centre, comprising 1104 patients with at least 3 years follow-up, found that the odds of a UTI increased 10-fold for those receiving botulinum detrusor injections [6].

### 5.4. Urodynamic Investigations

Urodynamic investigations are by far the most commonly used method for bladder evaluation, it is believed to increase the risk for UTIs, and therefore, the American Urological association suggests that high risk patients with neurogenic lower urinary tract dysfunction should receive antibiotic prophylaxis for UTI prophylaxis [72]. Data from a recent SCI study found that a history of UTI within the past 4 weeks prior to the urodynamic investigation increased the risk for a new UTI [73].

### 5.5. Bladder and Kidney Stones

Bladder and kidney stones are well-known risk factors for UTIs and evolve through two mechanisms; infection stones caused by urease-producing Gram-negative organisms and metabolic stones that passively trap bacteria from coexistent UTIs [74, 75]. By clinical experience, stones may stimulate the detrusor overactivity and hence the intravesical pressures, related to recurrent UTI's. In very rare occasion a foreign body, e.g. a hair, inserted through catheterisation, provides the basis for stone formation [76].

### 5.6. Postvoid Residual Urine Caused by Bladder Shapes

Merrit [77] originally described a correlation between postvoid residual volume and UTI frequency based on 105 SCI patients, which, however, could not be reproduced in a small-scale study in 12 SCI subjects [78]. Smaller volumes (<50 ml) in CIC-users did not predispose to UTIs [47]. Despite lack of clear evidence for a cut-off level, increased amounts of postvoid residual urine (>100 ml) is an accepted risk factor for UTI in the neurogenic population [62]. The reasons for experience residual urine could be many including anatomical bladder abnormalities, not proper education of patient, handling of IC catheter, and product choice (IC catheter).

Anatomical abnormalities that limit complete bladder emptying (e.g., prostatic impression, bladder diverticulum or trabeculated “Christmas tree bladders”) are conditions in which lagoons of urine are difficult to empty during catheterisation and so potentially provide a nidus for bacterial proliferation. There are no studies addressing these conditions, but it appears logical to accept such as risk factors. Such bladders may also have altered compliance, contributing to the UTI risk.

## 6. The Model: User Compliance/Adherence

In relation to UTI, compliance in this article describes the extent to which a CIC-catheter user adheres to the medical advice given by their treating health-care provider as to prevent UTIs [79] (compliance in this review does not consider reasons for discontinuing CIC management). As noted by Shekelle et al. [28], there was at that time a “paucity of data on the independent effect of psychosocial, behavioural, or hygienic factors on the risk of UTI in people with SCI”. Nothing seems to have changed substantially with respect to the evidence base of these factors, but it is generally recognized that factors like misconceptions, anxiety, embarrassment, and poor confidence can be barriers to CIC, which can be worsened by physical disabilities, e.g., dexterity or visual impairment [80, 81].

In the context of performing CIC it should also be taken into account that: (1) patients with neurodegenerative disorders often present with various levels of cognitive dysfunction; (2) psychiatric diagnoses, including depression and anxiety, are not unusual among individuals with relevant neurological diseases; (3) polypharmacy is not unusual in these patients and some medicines do impose a high anticholinergic burden. Cognitive function should be routinely assessed in neurourological patients to provide individualized counselling on IC technique.

### 6.1. Voiding Frequency

Voiding frequency and urine volume (two sides of the same coin) as infrequent voiding, result in larger bladder volumes that may over distend the bladder and so increase the risk for UTI. A survey of Canadian SCI patients found that voiding frequency was inversely related to number of UTIs, with one daily catheterisation having the highest UTI rate in a univariable analysis [25]. In a study of Paralympic athletes who catheterised from 1 to 10 per day (average 6 ± 2 times), the frequency of daily catheterisations was not related to the frequency of UTIs, *p*=0.07 [21]. As mentioned above, the catheterisation volume must be kept below 400 ml [45].

### 6.2. Fluid Intake

Poor fluid intake is generally considered as a risk [82] for UTIs in neurogenic bladder disease, and a low fluid intake has been associated with an increase in urine osmolality and acidity, which may predispose to UTIs [83]. In this context, the absence of diurnal variation of antidiuretic hormone levels in spinal cord injured subjects could play a role for adequate fluid intake [84]. All in all, empirical evidence for role of fluid intake for UTI prevention in the neurogenic population is scarce, and therefore, it is not possible to draw any firm evidence-based conclusions about daily fluid intake [83]. However, general medical experience supports the intake of daily adequate amounts of fluids, which recently was supported by a clinical study showing that increased water intake prevents recurrent cystitis in premenopausal women [85].

### 6.3. Nonhygienic Procedures

The Canadian survey of 935 SCI patients living in community determined CIC hygienic practices found, based on a univariate model, that genital and peritoneal cleaning was associated with reduced UTI rate as was self-catheterisation compared to catheterisation by others [25]. Wyndaele et al. [86] compared the results in 25 paraplegic patients started on CIC 35 days postinjury with 48 paraplegic patients catheterised postinjury by nurses with a nontouch technique and found comparable UTI rates in the two groups. The EAUN guidelines strictly encourages the no-touch techniques; however, no evidence for the no-touch technique on the UTI rate is given [81]. For comparison, a study was carried out on simulation models, evaluating the difference between classical sterile procedure and a no-touch procedure in hospital settings [87]. The no-touch procedure caused a lower amount of sterility errors and shorter duration of the intervention compared with the classical method. Whether less sterility errors translates into a lower rate of UTIs remains to be shown.

The basic importance of hand washing for prevention of infection was already emphasised by Semmelweis and Koch [88, 89]. The various guidelines also underline that patients who self-catheterise should disinfect or wash hands thoroughly with water and soap before catheterisation [82, 90–92]; however, it appears that the recommendations are based on experience from hospital setting and not on clinical evidence form community settings. A recent survey of self-reported habits amongst community dwelling SCI subjects showed that only half of the subjects washed hands as recommended, but no relationship was found with either occurrence or frequency of UTIs [93].

Obesity may be independently associated with UTIs [94, 95], particularly prominent in very obese men (BMI > 50) but less so for women. No data specific for the neurogenic population are available. It could be speculated that obese subjects have difficulty with bowel hygiene and as such are at increased risk of vulvovaginal symptoms and UTI.

Self-catheterisation versus catheterisation by others was associated with a significantly reduced UTI rate in a Canadian national survey of intermittent catheterisation practices following SCI [25]. Similar, after commencement of IC in a SB population, it appeared that IC self-catheterisation prompted a greater reduction of UTI than assisted IC [96].

### 6.4. Insufficient Education

Guidelines to IC recommend that comprehensive training on IC technique should be provided to neurourological patients [4, 78, 82], but adherence to CIC technique as recommended by health-care personal was just followed by 76% of responders [93]. A RCT examined the impact of an educational program on UTI frequencies [32]; the educational intervention resulted in less bacteriuria and a nonsignificant trend toward fewer symptom reports and antibiotic treatment episodes as well as decreased UTIs. However, for individuals with recurrent UTIs, increased knowledge may lead to increased perceptions of the severity of UTIs. Education for spinal cord injured athletes suggests that education may be advantageous [92] but a systematic review of educational programs did not detect a substantial beneficial effect [97]. A short-come of the previous studies did not assess quality of educational initiatives, as no objective feedback was obtained from the patients. In a European initiative undertaken by eight rehabilitation centres in Norway, France, and Italy, it was shown that a good education programme can improve adherence to IC during the first year at home (99 vs. 83% for the Education Programme and control groups, respectively; *p* < 0.05) [98]. From a clinical point of view, comprehensive training on IC technique should be provided to neurourological patients. Whenever a patient presents with UTI, IC technique and frequency must be reviewed [82].

### 6.5. Postvoid Residual Urine due to Incorrect Handling

The causes for residual urine could be many fold, as described previously. In particular, removal of the intermittent catheter before complete bladder emptying is frequently encountered in clinical practice and is considered as a risk for UTIs. This procedure should be included in the training of patients.

### 6.6. Residence Country and Social Support System (Reimbursement)

The differences in patterns of care are closely related to socioeconomic status and resources of the geographic area [44, 99]. CIC is well established in developed countries and seems to be part of standard patient care in the larger Asian and South American countries [100]. In the same way, low UTI incidence rates are related to developed countries, whereas higher rates are detected in less developed areas [21]. These findings underline the unmet needs present in many countries.

The importance of compliance and lifestyle for UTI prevention in community living subjects is not based on solid clinical evidence but rather driven by economic conditions, guideline recommendations, health-care personnel directions and patients own habits based on their individual disabilities and physical surroundings. The guidance given varies between institutions, regions, and countries, heavily influenced by social security systems and reimbursement schedules. Evaluation of IC regimen and individual costs related to IC should be done to improve nationwide control of cost. However, feasibility is difficult to evaluate and as cost.

## 7. The Model: Intermittent Catheters

### 7.1. Bacteria Inserted by Product and No Urethral Rinsing

Intermittent catheterisation *per se* is an important etiological risk factor for UTI in the neurogenic bladder [29]. It allows bacteria from the lower urethral region to be deposited directly into the bladder and cannot create the mechanical rinsing of the bladder that occurs during normal voiding. Additionally, nonhygienic intermittent catheterisation practices can introduce bacteria into the urinary tract. The use of no-touch catheter technique, which includes use of a urinary catheters without touch by the user's hand, like a nontouch sleeve and insertion tip has been shown to reduce the risk. Clinical studies suggest that use of a no-touch catheter is associated with a 30% reduction in bacteriuria and general low bacteriuria levels [16, 101]; however, data are based on few patients and bacterial counts only.

A hospital study reported on a no-touch catheter and technique with 35% less infection (UTIs not defined) per admission when compared to a retrospective, very different control group [102]. Both studies are therefore not providing clinical evidence for the benefit of a no-touch catheter system. In a 2 × 2 weeks crossover study of a new no-touch sleeve system compared to a conventional catheter, five UTIs were reported, but without information about in which group they occurred [103]. Interestingly, in the Canadian survey, there was no difference in UTI incidence rate if catheters were disinfected between use or not [25].

In summary, the positive influence of catheter design is controversial, but overall, the present evidence suggests beneficial use of hydrophilic catheters for CIC management.

### 7.2. Urethral and Bladder Trauma from Product

Chemical and physical properties of the catheter surface are important when looking at risk for creating trauma from product. Compared to gel lubrication, catheters with hydrophilic coatings caused significantly less urethral trauma (haematuria), less removal friction, and less pain [104]. This could be related to the clinical experience that lubricating catheters with a gel is insufficient to protect the urethra from injury, as the lubricant is lost upon entering the urethral meatus [105]. The osmolality of hydrophilic catheter coatings was found to reduce both removal friction and urethral trauma (haematuria) during catheterisation since a hyperosmolar coating appears gentler to the urethral mucosa due to its higher water content [106]. A study of urethral cytology in SCI patients performing CIC demonstrated a significantly lower inflammatory response in patients using hydrophilic catheters compared to uncoated catheters [107]. The data demonstrates that single-use hydrophilic catheters have properties that minimize urethral trauma.

Urethral strictures are a consequence of long-term use of CIC [6, 108]. The authors found during an observation period of 5-6 years a stricture rate of 25% and 19%, of which 36% and 21% required urethrotomy, respectively. Difficult and traumatic catheterisations may cause injuries ranging from a mucosal tear to more serious false passages, which are associated with UTIs and strictures and subsequently may require surgical management [109]; strictures may make future catheterisations difficult and cause repeated injury and UTIs [110]. Careful education of catheterisation technique and selection of the proper catheter is fundamental [111].

Patterns of use. The 2014 Cochrane review by Prieto et al. [112] “Intermittent catheterisation for long-term bladder management” was one of the leading documents concerning bladder management in the neurogenic population. The opinion expressed in the paper however concerned many clinicians and led to an independent reanalysis of the Cochrane data [113]; consequently, the Cochrane publication was withdrawn due to their erroneous data selection, data extraction, data analysis, and use of outdated UTI definition. The reanalysis could not detect any significant differences in the UTI incidence rates between aseptic vs. other techniques and single vs. multiple use of catheters due to the small number of participants and short or unclear duration of trials; it was not possible to draw final conclusions. In contrast to the Cochrane review, a significant difference favouring use of hydrophilic catheters vs. other catheters was detected.

The studies that were eligible for inclusion in the meta-analyses were executed in in-patient hospital/rehabilitation centres and/or community environments and included in total 502 patients, essentially evaluated over a few months (hospital/rehab centres: 209 patients during a median study period of 12 weeks; hospital/rehab and subsequent community residence: 123 SCI patients SCI during a 52-week study period; community: 56 SCI patients during 52 weeks and 82 SB children during a median study period of 16 weeks; 32 male patients with enlarged prostates were studied during a 6-week period). The two long-term trials of 52 weeks in SCI patients showed comparable results. De Ridder et al. [17] performed the trial in 123 patients during rehabilitation and subsequent community residence and found significantly less UTIs associated with hydrophilic catheters than with uncoated (single use). Cardenas et al. [20] in 56 community dwelling patients also documented significantly less UTIs associated with hydrophilic than with uncoated catheters (single use).

As stated by the authors of the meta-analysis, due to the inadequate numbers of participants and the predominantly short trial duration, additional trials are necessary to conclude about catheterisation technique and catheter usage. Based on the present level of evidence, the authors recommended single use hydrophilic catheters.

Since the analyses, based on data collected until 2014, several new studies have emerged. Rognoni et al. [114] also challenged the outcome of previously published meta-analyses and compared complication rates in terms of UTI and urethral trauma/haematuria related to hydrophilic coated catheters vs. nonhydrophilic urinary catheters. The meta-analyses exploring UTI frequencies showed a 16% lower risk ratio associated with hydrophilic catheters in comparison to standard ones (95% CI, 6–25%, *p*=0.003) and so corroborate the benefits of hydrophilic catheters.

Kiddoo et al. [115] studied the UTI incidence rate in a crossover trial (2 × 24 weeks) of single use hydrophilic catheters vs. multiple use polyvinylchloride catheters in 66 SP children. They found a statistically significant lower person week of UTI (defined as positive leucocyte dipstick and self-reported fever/pain/increased incontinence or cloudy/odorous urine) in multiple use, but no differences with respect to fever, antibiotic use visits to physicians, missed activities, and positive dipstick for leucocytes and haematuria. In other words, there was no difference in febrile or antibiotic-treated UTIs between use of these two catheter types.

### 7.3. Postvoid Residual Urine due to Product Design

Choosing the appropriate intermittent catheter tailored to the patient is important to avoid residual urine; this includes, e.g., choosing a catheter of a proper length with proper placement of the eyelets, considering catheter stiffness, and adapting catheter handling to this features.

## 8. Discussion

The purpose of this review was several folds: to remind clinicians that the correct diagnosis and thereby treatment/management of UTI/bacterial contamination in the ANLUTD patient population is challenging and far from simple; to update and discuss the UTI risk factors associated to IC in community settings; and to provide a simple, holistic, and useful risk factors model that can be used by the clinician for daily practice and thereby optimise the modification of these risk factors to the benefit of these patients.

A basic prerequisite is the alignment on the definition of a UTI. As described, many definitions are used in the real-world setting and complicate the understanding and comparability of results. Various medical societies have suggested their UTI definition, but it is imperative from a research perspective that a unified global UTI definition be agreed upon in order to objectively study current and future diagnostic and therapeutic treatment options for UTI.

Overall, the evidence for many of these risk factors role for the development of UTIs are limited and the impact of the personal experience of the clinicians may affect the handling of these risk factors, which should not prevent deciding on specific diagnosis, like UTI, based on available guidelines.

Some of the risk factors have with relative certainty causality roles for UTIs, like high intravesical pressure, the coexistence of a detrusor external-sphincter system dysfunction, catheter and catheterisation procedures, and nonadherence to CIC recommendations. Other proposed risk factors, like Botox injections, urodynamic investigations, or patient education, may to a higher degree be based on expert opinions. Future research is clearly necessary for a better understanding of the impact of a specific risk, both in terms of evaluating a direct causality as well as estimating their importance in real-life situations, where other factors additionally can modify the risk.

Individual conditions for each patient also play an important role and the clinicians should take all this individual variability into account. Each new UTI should trigger a holistic assessment of the patient's situation including overall health, mobility, bladder urodynamic situation, and bowel function. Further, the patients' cognitive function should be assessed to determine the correct understanding of IC procedures and compliance issues. Recurrent UTIs warrant additional investigation and assessment, which may involve imaging, flexible cystoscopy, and video-urodynamic assessment. In case of carer-assisted IC, procedure-related and educational approaches may be necessary.

The importance of catheter properties appears to be of relevance. The risk related to different IC-catheter types, like uncoated catheters, reuse catheters, or single use, hydrophilic ready-to-use catheters have been thoroughly discussed in the recent years; meta-analyses have provided some indications that hydrophilic catheters appear to be linked to the lowest UTI risk [113, 114]. This reflects the physiological common sense that less trauma to urethra and bladder is likely to leads to less UTI. Catheters ready-to-use (hydrophilic coated catheter) appear to cause less constraints for daily use in patients suffering from debilitating conditions like SCI, SB, or MS. Due to the heterogeneity of catheter-users, it can be anticipated, that a hydrophilic, ready-to-use catheter takes away the user's variability in use of the product. This product safety feature of ready-to-use catheters in essence improves product safety by decreasing individual variability in its use.

## 9. Conclusion

There is a need for alignment of the definition and diagnosis of UTI. It may be done disease per disease or more generally. Even with less complications than other bladder management methods, CIC still expose ANLUTD patients with a high risk of UTIs, a condition associated with increased morbidity and mortality in this patient group. There is a paucity of evidence describing the UTI risk profile, and well-designed clinical trials are warranted to provide the clinician a better platform for adequate management of the UTI risk profile to the benefit of these patients. Guidelines, when available, should be adhered to.

## Figures and Tables

**Table 1:** Definitions for catheter-associated urinary tract infection.

2017/18 Guidelines on Neuro-Urology of the European Association of Urology (EAU) [4]. +^*∗*^Blok et al. EAU guidelines on Neuro-Urology 2015 [40]	Signs and/or symptoms accompanied by laboratory findings of a UTI (bacteriuria, leucocyturia^a^, and positive urine culture). Significant bacteriuria in persons performing IC is present with >10^2^ colony-forming units (cfu)/mL, >10^4^ cfu/mL in clean-void specimens^*∗*^, and any detectable concentration in suprapubic aspirates. The most common signs and symptoms in those with neuro-urological disorders are fever, new onset or increase in incontinence, including leaking around an indwelling catheter, increased spasticity, malaise, lethargy or sense of unease, cloudy urine with increased urine odour, discomfort or pain over the kidney or bladder, dysuria, or autonomic dysreflexia

2009 International Clinical Practice Guidelines from the Infectious Diseases Society of America (IDSA) [41].	Symptoms or signs compatible with UTI with no other identified source of infection along with ≥10^3^ cfu/mL of ≥1 bacterial species in a single catheter urine specimen or in a midstream voided urine specimen from a patient whose urethral, suprapubic, or condom catheter has been removed within the previous 48 h^b^

ISCoS Urinary Tract Infection Basic Dataset [42].	(i) New onset of symptoms accompanied by laboratory findings (bacteriuria, leukocyturia and positive urine culture) of a UTI. (ii) Symptoms: fever, urinary incontinence/failure of control or leaking around the catheter, spasticity, malaise, lethargy or sense of unease, cloudy urine, malodorous urine, pyuria/leukocyturia, back pain, bladder pain, dysuria, autonomic dysrreflexia (AD). (iii) A clean-catch midstream technique from an immediately installed urine catheter. Any positive culture should be reported. The clinical Microbiological Laboratory (CML), 10^3^ CFU ml^−1^ is a reliable finding with standardized inoculation with 10 *µ*l urine.

^a^Leucocyturia is defined as 10 or more leucocytes in centrifuged urine samples per microscopic field (400×). ^b^In the catheterised patient, pyuria is not diagnostic of CA-bacteriuria or CAUTI, and the presence, absence, or degree of pyuria alone does not, by itself, differentiate catheter-associated asymptomatic bacteriuria from CAUTI. However, the absence of pyuria in a symptomatic catheterised patient suggests a diagnosis other than CAUTI.

## Appendix

Intermittent catheterisation (IC) is defined as drainage of the bladder or a urinary reservoir with subsequent removal of the catheter mostly at regular intervals. Clean IC (CIC): this involves use of a clean technique. This implies ordinary hand and genital washing techniques and use of disposable or cleansed reusable catheters [1].

  Aseptic IC: this implies genital antiseptic preparation and the use of sterile (single use) catheters and instruments/gloves in a designated clean area.
  Sterile IC: complete sterile setting, including genital skin antisepsis, sterile gloves, forceps, gown, and mask.
  No-touch technique IC: this was introduced as an easier way for the patient to perform self-intermittent catheterisation with a ready-to-use catheter (prelubricated catheter, usually a hydrophilic catheter).
